# Subchondral fracture of the femoral head after acetabular fracture: a case report

**DOI:** 10.1186/1752-1947-8-447

**Published:** 2014-12-19

**Authors:** Keiichiro Iida, Satoshi Hamai, Takuaki Yamamoto, Yasuharu Nakashima, Goro Motomura, Masanobu Ohishi, Kazuyuki Karasuyama, Yukihide Iwamoto

**Affiliations:** Department of Orthopaedic Surgery, Faculty of Medical Sciences, Kyushu University, 3-1-1 Maidashi, Higashi-ku, Fukuoka, 812-8582 Japan

**Keywords:** Acetabular fracture, Collapse of the femoral head, Subchondral fracture of the femoral head, Post-traumatic osteoarthritis

## Abstract

**Introduction:**

Preventing post-traumatic osteoarthritis is a challenging problem following acetabular fracture. Progressive osteoarthritis is considered to be caused by an irregular articular surface of the acetabular roof or cartilage injury, but little is known about the pathogenesis of collapse of the femoral head after acetabular fracture. We report a case of post-traumatic osteoarthritis after acetabular fracture in which subchondral fracture of the femoral head contributed to the progressive collapse of the femoral head and osteoarthritis. To the best of our knowledge, there has been no previous report of subchondral fracture of the femoral head after acetabular fracture.

**Case presentation:**

A 58-year-old Japanese man fell from a ladder. He was diagnosed with a left acetabular fracture, which was managed conservatively. He developed left coxalgia six months after injury and was seen at our institution one year after the onset of pain. The left acetabular fracture had fused, but his left femoral head had collapsed. The images at the time of injury showed a fracture of the acetabular roof, and an approximately 2mm step-off existed in the articular surface. Retrospective evaluation of the plain radiographs and computed tomography images showed that his femoral head had progressively collapsed. Our patient underwent total hip arthroplasty. Histopathologic findings demonstrated that the collapse of his femoral head was caused by a subchondral fracture of his femoral head.

**Conclusion:**

Our experience with this case indicates that in addition to post-traumatic osteonecrosis, subchondral fracture may need to be considered in cases with progressive collapse of the femoral head after acetabular fracture.

## Introduction

One of the major goals in managing patients with acetabular fractures is to prevent post-traumatic osteoarthritis. The stability and concentricity of the hip joint and the condition of both the acetabular roof and femoral head are important factors in achieving a good outcome [1, 2]. Insufficient reduction of the articular surface leads to osteoarthritis due to increased stress on the articular cartilage, and anatomical reduction and internal fixation are required in patients with displaced fractures of the acetabulum [3]. In many cases, post-traumatic osteoarthritis develops early after acetabular fractures [3]. Specifically, wear on the femoral head after an acetabular fracture is hypothesized to induce early osteoarthritis, which can progress to such a degree that it is referred to as a major complication [3–5]. The irregular articular surface of the acetabulum and/or damage to the femoral head at the time of injury is thought to cause the progressive wear and rapid collapse of the femoral head [3, 6]. Such collapse is a more frequent complication than collapse resulting from osteonecrosis [3]; however, the detailed pathogenesis underlying collapse of the femoral head has not been elucidated.

We present a case of post-traumatic osteoarthritis after an acetabular fracture in which a subchondral fracture of the femoral head contributed to the progressive collapse of the femoral head and the development of osteoarthritis.

## Case presentation

A 58-year-old Japanese man fell from a ladder and sustained a left acetabular fracture. His injury was managed conservatively by a previous physician and he was able to walk without pain. He developed left coxalgia six months after the injury, and was unable to walk when he visited our institution, one year after the onset of pain. He had a past history of hepatic cirrhosis due to hepatitis C and had undergone a living liver transplantation 12 years prior to the current admission. He had consumed no alcohol since the liver transplantation and had no history of corticosteroid therapy.

**Figure 1:**
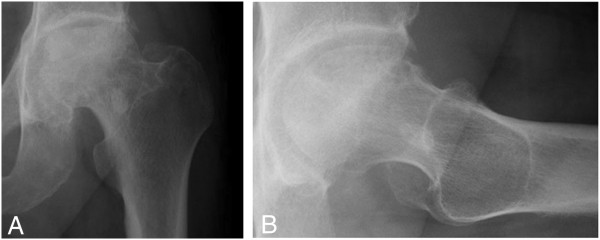
**Plain radiographs show a fused acetabular fracture and collapsed left femoral head. (A)** Anteroposterior and **(B)** lateral plain radiographs of the left hip obtained a year and a half after the left acetabular fracture show collapse in the anterosuperior portion of the left femoral head with joint space narrowing.

**Figure 2:**
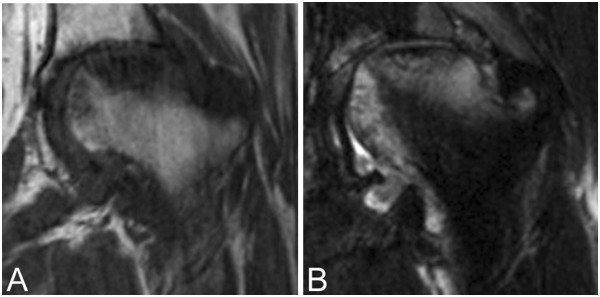
**Magnetic resonance images show a bone marrow edema pattern. (A)** Coronal T1-weighted magnetic resonance (MR) image shows a diffuse low-intensity signal in the proximal portion of the femoral head. **(B)** T2-weighted fat-suppression MR image shows diffuse high-intensity signals in both the proximal and medial portions of the femoral head.

**Figure 3:**
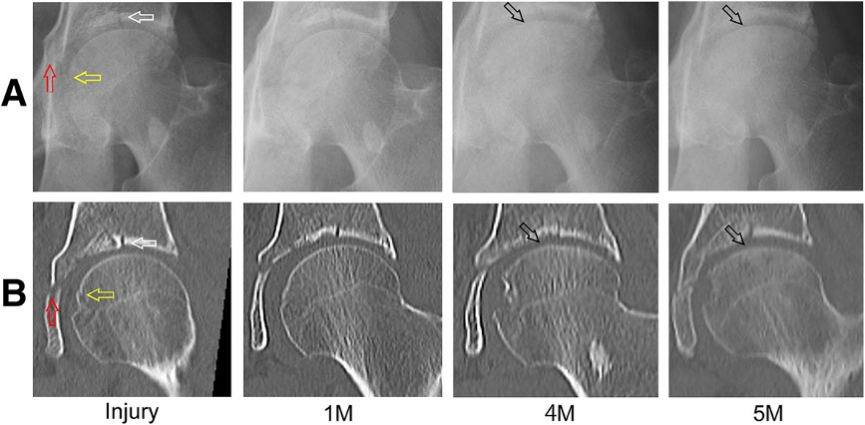
**The weight-bearing area of the femoral head shows progressive collapse. (A)** Anteroposterior plain radiographs and **(B)** coronal computed tomography (CT) images of the left hip at the time of injury (Injury) show a fracture in the acetabular roof (white arrow), inner wall of the acetabulum (red arrow), and medial articular surface of the femoral head (yellow arrow). The series of images (one month (1M), four months (4M) and five months (5M) after injury) show the progression of collapse in the femoral head (black arrows), with residual displacement of the fracture in the acetabular roof.

Plain radiography and computed tomography (CT) images obtained at his first visit to our institution showed a fused acetabular fracture and collapsed left femoral head (Figure 1). Magnetic resonance (MR) imaging demonstrated a low-intensity area on T1-weighted images consistent with the collapsed area (Figure 2). Fat-suppressed T2-weighted images demonstrated a diffuse region of high intensity in the proximal and medial portions of his femoral head. The initial images obtained at the time of injury showed fractures in the acetabular roof and inner wall of his acetabulum without hip dislocation, and an approximate 2mm step-off was observed on the articular surface of the acetabular roof (Figure 3). The acetabular fracture was classified as an anterior column-posterior hemitransverse fracture according to the Judet-Letournel classification system [7]. There was also a fracture on the medial articular surface of his femoral head.

**Figure 4:**
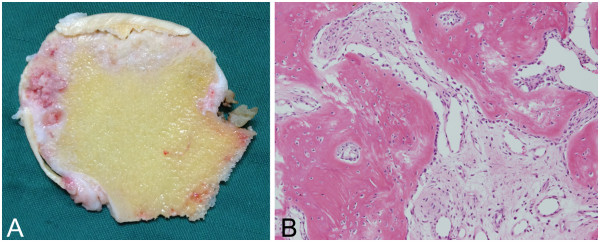
**Histological findings show a subchondral fracture of the femoral head and no evidence of antecedent osteonecrosis. (A)** Mid-coronal cut section of the resected left femoral head shows a linear fracture line paralleling the subchondral bone endplate. **(B)** The photomicrograph obtained from the subchondral fractured lesion shows marked fracture callus and vascular rich granulation tissue (hematoxylin and eosin, ×100).

Retrospective revaluation of the plain radiographs and CT images revealed that the weight-bearing area of his femoral head had progressively collapsed, with residual displacement of the acetabular fracture (Figure 3). Dual X-ray absorptiometry showed that our patient’s bone mineral density was 0.657g/cm^2^ in the unaffected right femoral neck, which was 76% of the young adult mean, indicating the presence of osteopenia. Our patient underwent left total hip arthroplasty for advanced osteoarthritis associated with the collapse of his femoral head. The resected femoral head exhibited a flattened widespread surface with a flap of articular cartilage and subchondral bone, and the cut section demonstrated a subchondral fracture line parallel to the articular surface (Figure 4A). Histological examination demonstrated repair tissue consisting of marked fracture callus and vascular rich granulation tissue on both sides of the fracture line (Figure 4B). There was no evidence of antecedent osteonecrosis. Based on these histopathologic findings, we determined that the collapsed lesion was caused by a subchondral fracture of his femoral head resulting from acetabular fracture.

## Discussion

Both osteonecrosis and subchondral insufficiency fractures are known to cause collapse of the femoral head [8, 9]. Post-traumatic osteonecrosis is a frequent complication after femoral head dislocation, whereas the occurrence of a subchondral fracture after an acetabular fracture has not yet been reported. We herein propose that subchondral fractures may have occurred in cases showing a collapsed lesion in the femoral head after an acetabular fracture.

Post-traumatic osteoarthritis is a major problem in patients with acetabular fractures [3]. Tannast *et al*. reported that 21% of patients with operatively treated displaced acetabular fractures required total hip arthroplasty within 20 years [6]. The progression of osteoarthritis is so rapid that in many cases total hip arthroplasty is required within one or two years after the acetabular fracture [3, 6]. The osteoarthritis observed in such cases is caused by wear on the femoral head due to increased friction during joint motion and elevated pressure on the articular cartilage resulting from an irregular articular surface [5, 10]. Displacement of the articular surface of the acetabular roof should be less than 2mm to prevent subsequent osteoarthritis [1]. When the step-off of the acetabular roof exceeds the thickness of the cartilage, the ability of the hyaline cartilage to compensate reaches its limit, and the articular surface cannot be repaired [5, 11]. The 2mm step-off of the acetabular roof observed in our case was at the borderline of the criterion, and we believe that subsequent increased mechanical stress on the weight-bearing area induced progressive osteoarthritis with collapse of the femoral head.

Subchondral fractures of the femoral head have been associated with insufficiency fractures [9, 12]. Subchondral insufficiency fractures of the femoral head generally occur in elderly patients with osteoporosis and characteristically present with acute pain in the hip without any obvious antecedent trauma [13, 14]. Subchondral fractures can also occur in young adults with normal bone quality in whom the joints are subjected to high levels of repetitive stress, as observed in patients with fatigue fractures [15]. Because the clinical features and radiological findings of subchondral insufficiency fractures of the femoral head resemble those of osteonecrosis, the differential diagnosis is sometimes difficult [14]. In our case, a subchondral fracture was diagnosed based on our patient’s clinical features of progressive destruction of his femoral head, high-intensity signal of the proximal segment on fat-suppressed T2-weighted MR images, timing of the onset of pain after the acetabular fracture, and histological findings. The histological findings showed repair tissue consisting of marked callus tissue and the formation of granulation tissue on both sides of the fracture without any evidence of antecedent osteonecrosis. We did not use the word ‘insufficiency’ here because the subchondral fracture was obviously caused by the acetabular fracture. However, it is worth considering that our patient’s bone quality may have played a role in the onset of the subchondral fracture. It is reasonable to suggest that the subchondral fracture developed because of increased mechanical stress on his femoral head, with underlying osteopenia.

Damage to the femoral head is the factor that most clearly predicts a poor prognosis after acetabular fracture [3, 16], and the presence of a fracture in the weight-bearing area of the femoral head is a significant risk factor for collapse [17]. We do not believe that the initial injury to the medial articular surface of our patient’s femoral head contributed to his prognosis of osteoarthritis, as it was not located in a weight-bearing area. However, we cannot deny the possibility of an occult fracture in his femoral head at the time of injury, which would have been detectable only on MR imaging. Potter *et al*. reported that damage to the femoral head occurs as the head impacts the acetabulum at the time of injury, and documented 24 cases of subchondral contusion of the femoral head on MR images in which the CT findings were normal [18]. As soon as the patient’s general condition allows, MR imaging should be performed to rule out occult injuries of the femoral head, including subchondral fractures, to permit a prompt diagnosis and accurate treatment.

## Conclusions

To the best of our knowledge, this is the first case report of a subchondral fracture of the femoral head occurring after an acetabular fracture. Collapse of the femoral head was found to be caused by the subchondral fracture based on histological findings. Increased stress on the femoral head due to the irregular articular surface was considered to be the causative factor of the subchondral fracture. It is important to recognize that subchondral fractures of the femoral head can occur in patients with a history of acetabular fractures.

## Consent

Written informed consent was obtained from the patient for publication of this case report and any accompanying images. A copy of the written consent is available for review by the Editor-in-Chief of this journal.
